# The Role of Metagenomics in Precision Nutrition

**DOI:** 10.3390/nu12061668

**Published:** 2020-06-04

**Authors:** Katsumi Iizuka, Daisuke Yabe

**Affiliations:** 1 Department of Diabetes and Endocrinology, Gifu University Graduate School of Medicine, Gifu 501-1194, Japan; ydaisuke-kyoto@umin.ac.jp; 2 Yutaka Seino Distinguished Center for Diabetes Research, Kansai Electric Power Medical Research Institute, Kobe 650-0017, Japan; 3 Division of Molecular and Metabolic Medicine, Department of Physiology and Cell Biology, Kobe University Graduate School of Medicine, Kobe 650-0017, Japan

**Keywords:** metagenomics, gut microbiome, nutrigenomics, nutrigenetics, precision nutrition, ChREBP

Conventional recommendations for dietary intervention have been generally based on population groups divided by gender and age. However, it is well known that diversities in lifestyle, culture, social status, and genetic variants affect responses to nutritional intervention. Therefore, the concept of precision nutrition has been advocated. Precision nutrition refers to individual nutritional regimens designed to treat or prevent various diseases. To plan precision nutrition, the interplay between metabolic, genetic, social, and environmental factors must be considered [1]. Precision nutrition can be realized at three levels: (1) dietary advice based on general guidelines; (2) personalized nutrition based on phenotypes and laboratory tests (anthropometry, biochemical and metabolic analysis, physical activity); and (3) genotype-directed nutrition [1]. Recently, to promote precision nutrition, nutrigenetics and nutrigenomics have become favored tools. An example of nutrigenetic analysis is clarification of the associations between nutritional response and genetic variants such as single nucleotide polymorphisms. In contrast, nutrigenomics shows how dietary factors influence gene expression, and thereby, affect protein and metabolite levels [1]. Regarding nutrigenomics, metagenomics that focus on studies of the gut microbiome have received much attention in recent years [1]. The gut microbiome has been referred to as another organ, and microbiota have been shown to be capable of altering gene expression and protein synthesis to produce functional metabolites [2]. Interestingly, the diversity of gut bacteria found in healthy individuals is known to be radically altered in certain diseases, triggering inflammation and leading to diabetes and inflammatory bowel disease [2]. It has also been reported that transplantation of stool from healthy people can improve the disease condition. Thus, microbiome and genetic variation are important factors in facilitating precise nutrition.

A recent review published in *Nutrients* focused on the relationships between precision nutrition and the microbiome [2]. The authors summarized the development of gut microbiota from birth and during aging. The diversity of gut microbiota increases with age to the first year, and establishment of an adult-like microbiome occurs between 2 and 5 years of age and is dominated by Firmicutes and Bacteroides. The enterotype concept holds that Firmicutes- and Bacteroides-dominant patterns are linked to long-term carbohydrate (fiber) and animal fat and/or protein rich diet, respectively [2,3]. Furthermore, in elderly subjects, microbial diversity decreases with decreased microbial production of butyrate. The authors then describe the roles of the microbiome in the production of bioactive metabolites (short chain fatty acids, essential vitamins, secondary bile acid, neurochemicals), colonization resistance, immunity, and mucosal integrity. Moreover, intake of westernized diets that are rich in animal protein, saturated fatty acid, sugar, alcohol, and corn-derived fructose promote low microbial diversity and are linked to increased risk of IBD, cancer, liver disease, and recurrent C. difficile infection. They further describe the relationship between microbial metabolites (short chain fatty acids, trimethylamine, p-cresol, indoxyl sulfate, branched chain amino acids) and metabolic diseases [2]. They also discuss the effects of diet type and dietary components (carbohydrate, fat, and protein) on gut microbiota [2]. 

Following their review, we can understand that metagenomics is a useful tool to facilitate precise nutrition, but there remain problems involved in the interpretation of metagenomics data. While the enterotype concept is attractive for its ability to predict biomarkers of responsiveness to dietary intervention, there is the possibility that factors other than population, age and diet groups, such as genetic variation and medication, underlie the enterotypes. For example, carbohydrate response element binding protein (ChREBP) regulates intestinal glucose and fructose absorption via gene transcription of *Glut5* and *Ketohexokinase* [4], thereby decreasing fructose absorption in ChREBP^-/-^ mice [4]. We previously reported that genetic deletion of ChREBP and medication by miglitol, a sucrase isomaltase inhibitor, affects gut microbiome composition and induces irritable bowel syndrome [5]. These findings suggested that genetic variants affecting intestinal nutrient absorption might affect composition of the microbiome. Moreover, initial studies reported differences in community gut microbial diversity and in the Bacteroidetes/Firmicutes (B/F) ratio among obese and non-obese individuals [3], while meta-analysis did not reveal a significant difference in B/F ratio between the lean and obese groups [6]. Falony et al. found an association between microbiome composition and BMI when analyzing the gut microbiome in 1106 subjects from the Flemish Gut Flora Project (FGFP) [6,7]. Importantly, they found that the use of medication showed the largest explanatory value for microbiome variation as demonstrated in our study [7]. These findings clearly show that interactions between gut microbiota and factors such as age, genetic variants, medications, and food preferences should be taken into account when interpreting metagenomics data. Second, gut microbiota-derived metabolites have both beneficial and detrimental effects. For example, beneficial roles of acetate on body weight are based on appetite suppression [8]. In contrast, detrimental effects of acetate are based on increased insulin secretion and increased supply of the substrate for hepatic lipogenesis [9,10]. We should therefore consider both beneficial and detrimental aspects when modulating gut microbiota-derived metabolites for precise nutrition. In conclusion, metagenomics is a promising tool to enable personalized nutrition; integration of metagenomics and other -omics using “big data” is needed for accurate interpretation of metagenomic information.
